# Perforated Emphysematous Cholecystitis: A Race Against Time

**DOI:** 10.7759/cureus.35123

**Published:** 2023-02-17

**Authors:** Ashminie P Misir, Ilma Vahora, Gabrielle Unbehaun, Chandni Patel, Frederick Tiesenga

**Affiliations:** 1 General Surgery, St. George's University School of Medicine, Chicago, USA; 2 General Surgery, West Suburban Medical Center, Chicago, USA

**Keywords:** gallbladder stones, necrotic gallbladder, open cholecystectomy, emphysematous cholecystitis, perforated gallbladder

## Abstract

Emphysematous cholecystitis is a rare infection of the gallbladder that stems from acute cholecystitis. It can rapidly progress and perforate the gallbladder, which would require urgent surgical intervention. A perforated gallbladder can be diagnosed using an abdominal computed tomography by confirming the presence of air in the gallbladder lumen with adjacent extraluminal air. The causes of ruptured emphysematous cholecystitis include, but are not limited to, diabetes, atherosclerotic changes in blood vessels, and infection with *Clostridium perfringens, Escherichia coli, and Klebsiella spp.*, and is usually present in diabetic men. We report on a 57-year-old female who developed gall bladder perforation with an overflow of gallstones into the peritoneum without a history of diabetes or atherosclerotic disease. Due to the vast availability of computerized tomography and early surgical intervention, the rate of mortality due to perforated emphysematous cholecystitis has decreased over the last few decades.

## Introduction

Classic signs of acute cholecystitis can often lead to a more severe diagnosis upon radiologic indications such as a perforated emphysematous gallbladder. Also known as clostridial cholecystitis, this condition develops in 1% of the patients that are diagnosed with all cases of acute cholecystitis and is associated with high mortality (around 15%) due to perforation, which will be discussed in this patient’s case [1-3]. Imaging studies are especially important in this case, as it identifies features of the gallbladder that classified this surgery as emergent. The causes of an emphysematous gallbladder can range from diabetes, atherosclerotic disease, or infection from bacteria such as *Clostridium perfringens, Escherichia coli, and Klebsiella spp.* [1-4]. This case report will further examine the presenting signs and symptoms of a perforated emphysematous gallbladder to correlate future cases of a similar nature.

## Case presentation

A 57-year-old female presented to the emergency department with complaints of generalized, but primarily upper, abdominal pain, nausea, and vomiting for two days prior to coming to the emergency department. She denied fever, chills, or diarrhea. Her past medical history is significant for breast cancer. The patient is a nonsmoker with a surgical history of left mastectomy with reconstruction 20 years ago, abdominoplasty, and a colonoscopy completed within the last five months, which showed impressions of a single non-bleeding diverticulum with a small opening in the ascending colon.

**Figure 1:**
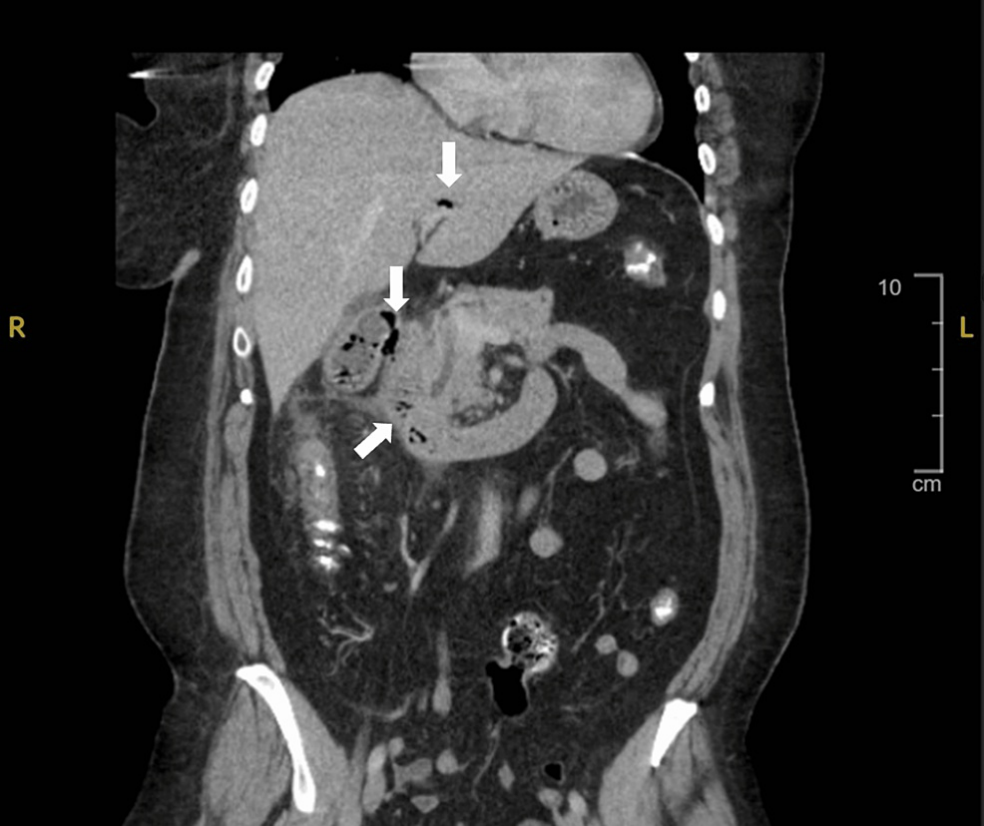
Computerized tomography with IV contrast of the abdomen and pelvis, coronal view depicting pneumobilia (free air) in the biliary system, gallbladder, and duodenum (depicted by the arrows)

**Figure 2:**
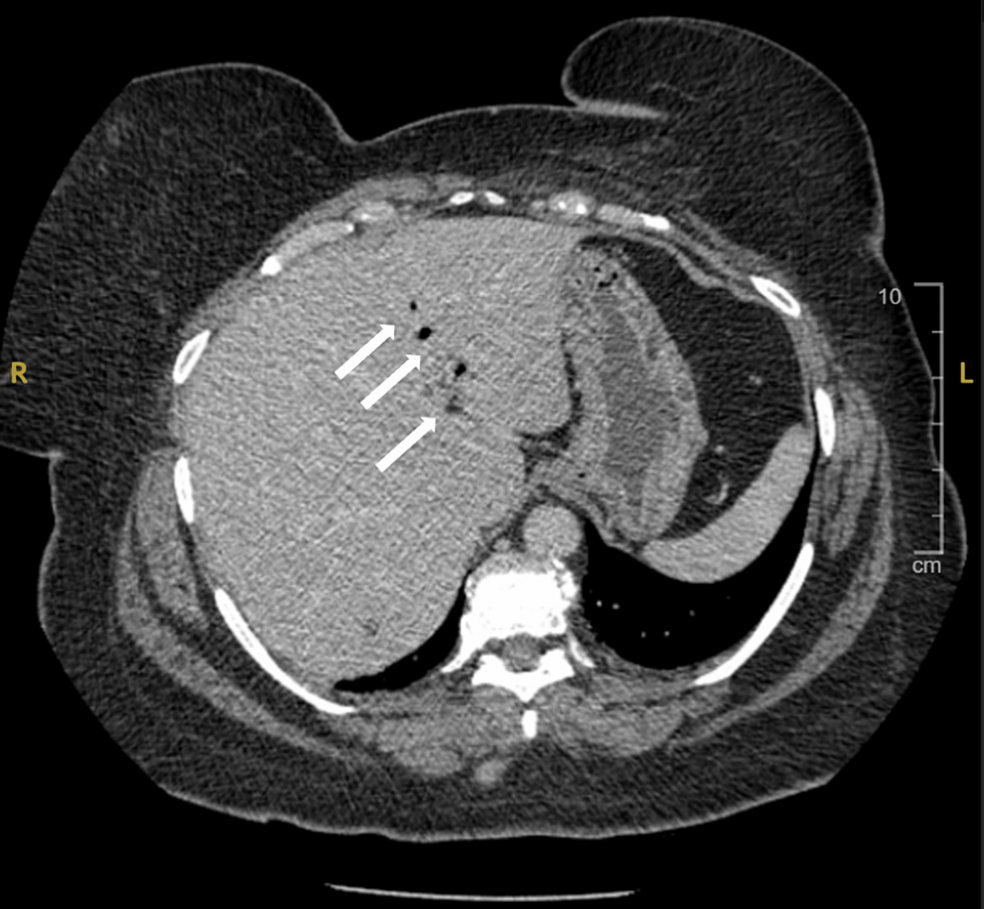
Computerized tomography with IV contrast of the abdomen and pelvis, axial view displaying pneumobilia (arrows) in the biliary system

**Figure 3:**
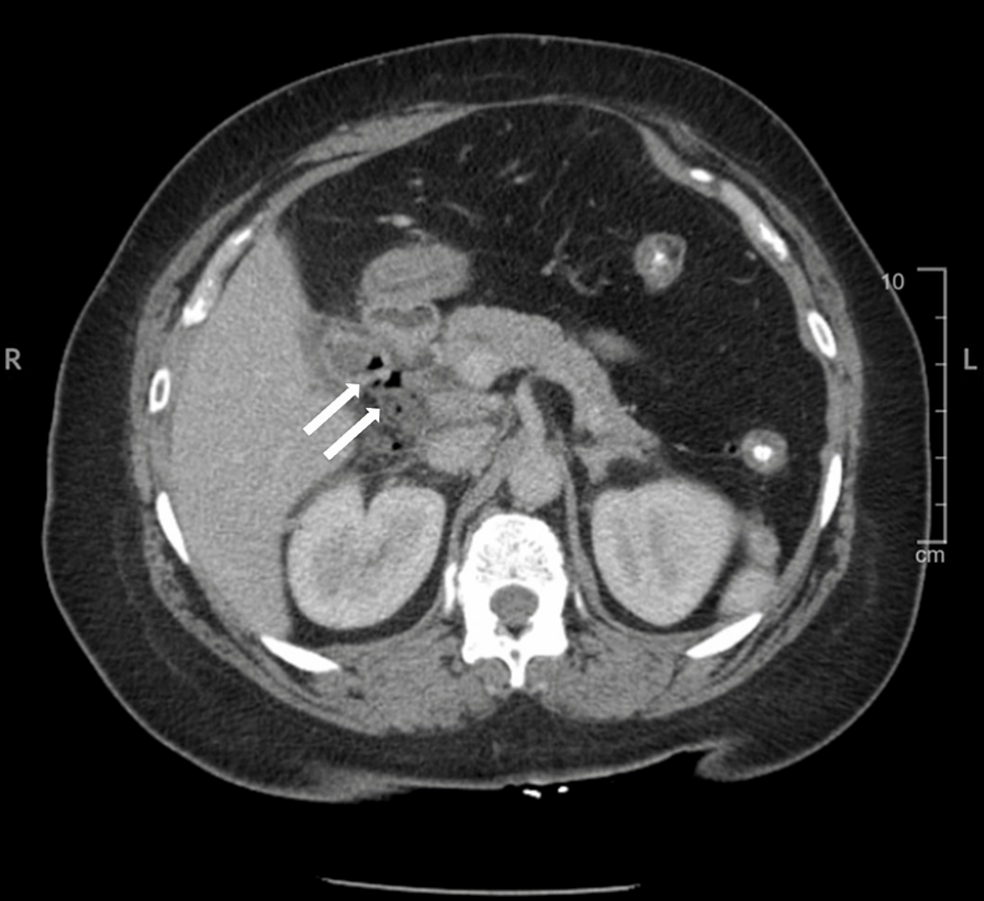
Computerized tomography with IV contrast of the abdomen and pelvis, supine position, displaying pneumobilia

**Figure 4:**
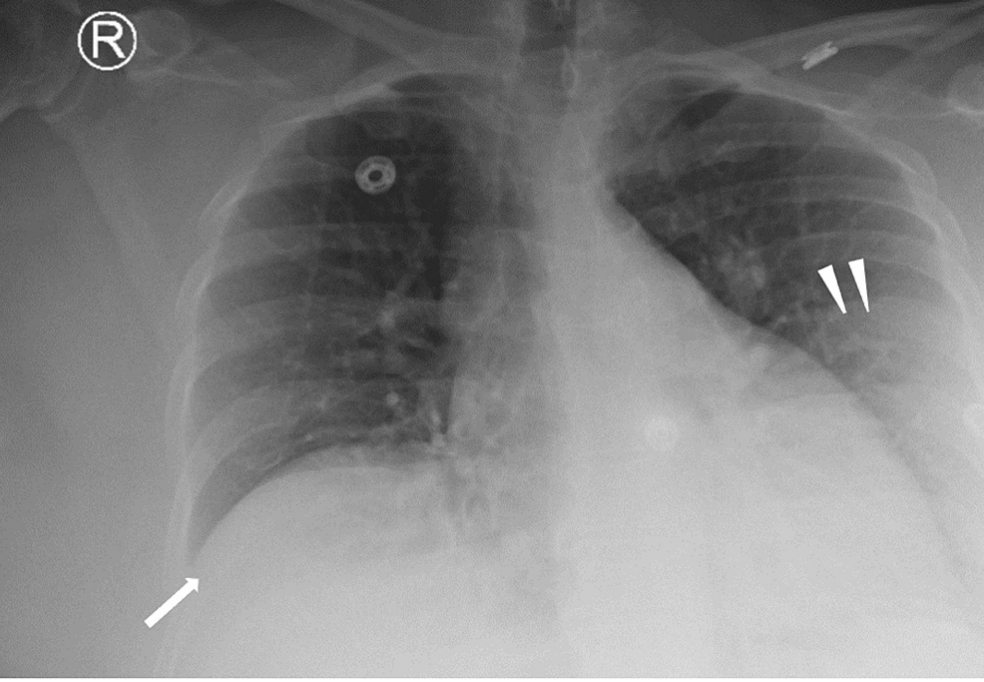
Chest X-ray displaying a mild elevation of the hemidiaphragm (arrows) Haziness along the lateral left mid to lower lung field is artifactual secondary to overlying soft tissue (arrowheads).

Upon arrival, the patient was afebrile with vital signs of borderline tachycardia with a heart rate of 99 BPM, tachypneic respiratory rate of 22 BPM, a blood pressure of 103/55 mmHg, and SPO_2_ of 97% on room air. On examination, the abdomen was soft but tender to palpation diffusely, more so in the upper right quadrant and epigastric area, with a positive Murphy sign. The patient was negative for McBurney’s point tenderness and no distension or rebound guarding was found. Blood and urine taken on the day of admission showed leukocytosis of 15.5 g/dl and normal hemoglobin and hematocrit of 13.2 g/dl and 42.5 g/dl, respectively. Her liver enzymes and bilirubin levels were within normal limits. The patient's lab values can be seen in Table 1. A computed tomography scan of the abdomen and pelvis showed impressions consistent with an emphysematous gallbladder with small perforation and possible fistula to the duodenum. Findings of pneumobilia showing free air in the biliary system are evident in Figures 1-3. A chest X-ray indicated a mild elevation of the right hemidiaphragm, as seen in Figure 4. Based on the imaging studies, the emergency department sent a consult request to the general surgery department.

**Table 1:** Preoperative and postoperative laboratory parameters MRSA: methicillin-resistant Staphylococcus aureus

Data	Day of admission	Post-op Day 1	Post-op Day 2	Post-op Day 3
Leukocyte count (million/mm^3)* (Ref: 4.5-10.5 million/mm^3)*	15.5	12.0	11.4	7.4
Erythrocyte count (million/mm^3) *(Ref: 3.6- 5 million/mm^3)*	5.57	4.91	4.82	4.64
Hemoglobin, blood (g/dl)* (Ref: 12.1 - 15.1 g/dL)*	13.2	11.9	11.5	11.0
Hematocrit (%) *(Ref: 36-48%)*	42.5	38.1	37.2	35.2
Sodium Level (mEq/L)* (Ref: 135-145 mEq/L)*	139	140	141	142
Potassium Level (mEq/L) *(Ref: 3.5-5.3 mEq/L)*	3.7	3.6	3.2	3.3
Chloride Level (mEq/L)* (Ref: 98-107 mEq/L)*	104	110	109	111
Carbon Dioxide (mm Hg)* (Ref: 23-29 mm Hg)*	22	21	21	20
Alkaline Phosphatase (U/L) *(Ref: 44-147 U/L)*	94	73	79	93
Alanine Aminotransferase (U/L) *(Ref: 4-36 U/L)*	13	44	39	46
Blood Urea Nitrogen (mg/dL) *(Ref: 6-24 mg/dL)*	12	10	11	13
Glucose (mg/dL)* (Ref: 70-100 mg/dL)*	157	163	125	103
Creatinine Level (mg/dL)* (Ref: 0.6-1.3 mg/dL)*	0.81	0.71	0.64	0.62
Calcium Level (mg/dL) *(Ref: 8.5-10.2 mg/dL)*	9.8	8.9	9.4	9.5
Protein Total (g/dL)* (Ref: 6.0-8.3 g/dL)*	7.2	6.2	6.2	6.2
Albumin Level (g/dL) *(Ref: 3.4-5.4 g/dL)*	2.9	2.2	2.0	1.9
Bilirubin Total (mg/dL) *(Ref: 0.1-1.2 mg/dL)*	0.7	0.9	0.8	0.8
Anion Gap (mEq/L) *(Ref: 4-12 mEq/L)*	13	9	11	11
BUN/Creatinine *(Ref: 12-20)*	14.8	14.1	17.2	21.0
Lactate (Venous) *(Ref: 0.5-2.2 mmol/L)*	2.6	1.3	-	-
Urinalysis Color (*determined by direct visual observation)*	Yellow			
Urinalysis pH* (Ref: 5.0-7.0)*	6.0			
Urinalysis Specific Gravity *(Ref: 1.005-1.035)*	1.025			
Urinalysis Glucose *(Ref: Negative)*	Negative			
Urinalysis Bilirubin *(Ref: Negative)*	Negative			
Urinalysis Ketones *(Ref: Negative)*	15 (A)			
Urinalysis Blood* (Ref: Negative)*	Large (A)			
Urinalysis Protein *(Ref: 10-20 mg/dL)*	30 (A)			
Urinalysis Urobilinogen (mg/dl) *(Ref: <2.0 mg/dL)*	4.0 (H)			
Urinalysis Nitrite* (Ref: Negative)*	Positive			
Urinalysis Leukocyte Esterase *(Ref: Negative)*	Negative			
MRSA Cultures *(Ref: Negative)*	Negative			
COVID-19 Polymerase Chain Reaction *(Ref: Negative)*	Negative			

Upon examination by the surgery team, the patient reported pain in the right upper quadrant when palpated. Murphy’s sign was positive. A decision was made to undergo urgent open cholecystectomy to which the patient consented. The patient was taken to the operative suite, a right upper quadrant Kocher incision was made to enter the peritoneal cavity, and the gallbladder was identified, which was found to be ruptured into the omentum and necrotic in appearance. The gallbladder was then dissected and sent to pathology, and cultures were taken. Antibiotic irrigation was utilized, a Jackson-Pratt drain was placed, and the operation was completed. Estimated blood loss during the procedure was minimal with an operating time of approximately 48 minutes. The patient was transferred to the intensive care unit for postoperative care. Three days after the operation, the patient was stable, tolerating a clear liquid diet, and was discharged from the intensive care unit to the family medicine inpatient unit; the Jackson-Pratt drain was removed on postoperative day four. However, the patient had an elevated aspartate aminotransferase (AST) of 157 and aspartate aminotransferase (ALT) of 196 five days post-surgery. The GI department was consulted. A hepatobiliary iminodiacetic acid (HIDA) scan, magnetic resonance cholangiopancreatography (MRCP), and abdominal MRI without IV contrast were within normal limits and the viral hepatitis panel was negative. Her liver function tests (LFTs) were stabilized and she was cleared from the GI department's standpoint. The patient was discharged to follow up for outpatient care for repeat LFTs with a 14-day course of cefuroxime and Flagyl.

**Figure 5:**
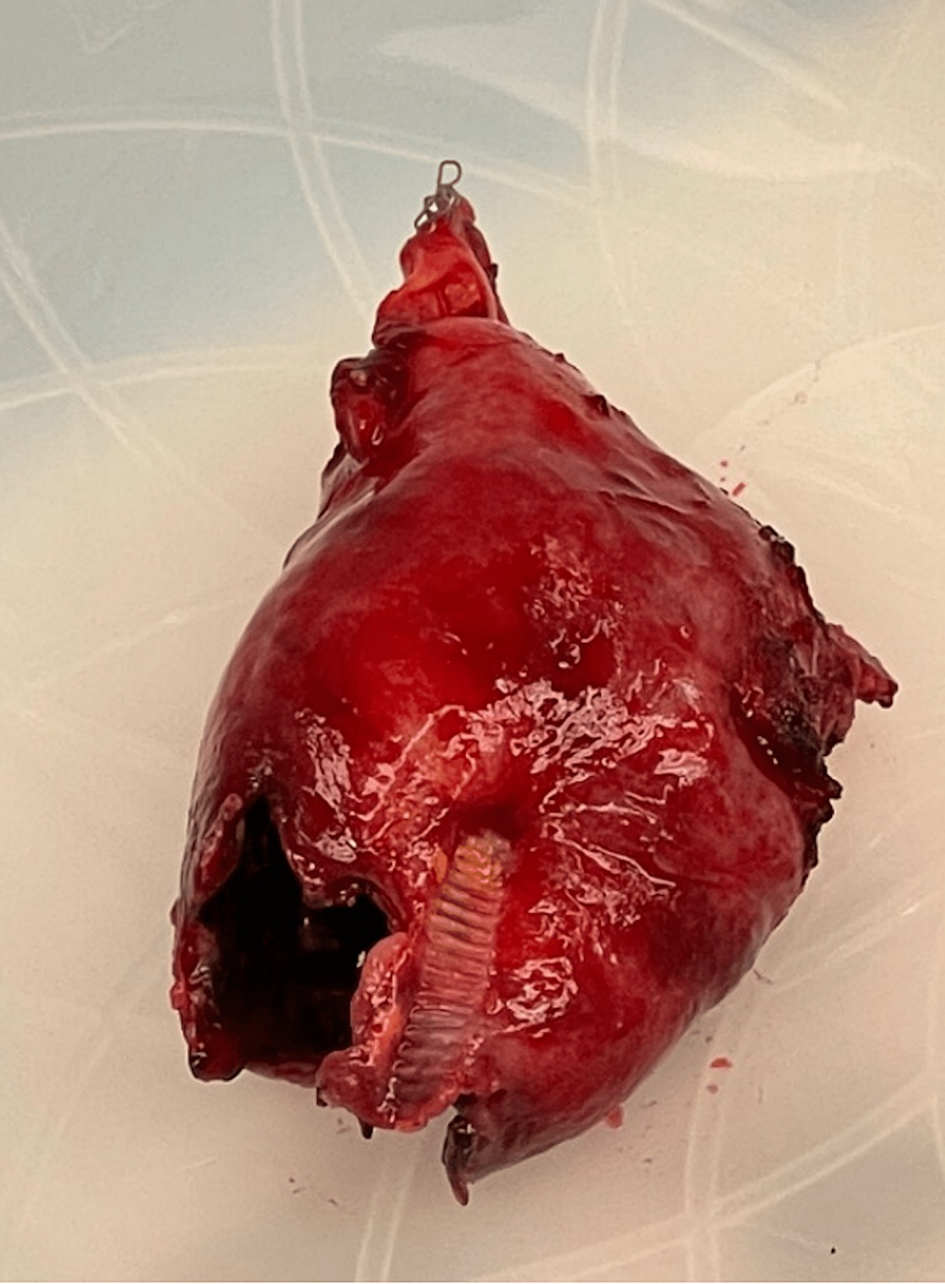
Perforated gallbladder, anterior view, post open cholecystectomy

The gallbladder that was removed during the operation can be seen in Figure 5. Pathology impressions of the gallbladder showed florid acute cholecystitis with multiple foci of mucosal ulcerations with an inflammatory process involving nearly the entire wall of the gallbladder wall and evidence of chronic cholecystitis with cholelithiasis. Culture results indicated mixed anaerobic organisms, with none predominating. Written informed consent was obtained from the patient for publication of this case report.

## Discussion

Emphysematous cholecystitis is a fulminant form of acute cholecystitis associated with an increased risk of gallbladder perforation and mortality due to differences in etiology and pathophysiology. While the clinical presentation of emphysematous cholecystitis is nearly indistinguishable from acute cholecystitis, the mortality rate of emphysematous cholecystitis is approximately 15% compared to that of uncomplicated acute cholecystitis at 1.4% [1-3], illuminating the importance of prompt diagnosis and intervention.

Emphysematous cholecystitis patients often have a history of vascular compromise, predisposing their gallbladder to ischemia, and diabetes mellitus, creating a microenvironment supporting anaerobic bacterial overgrowth [1-5]. Emphysematous cholecystitis is most commonly caused by anaerobic organisms, such as *Clostridia spp., Klebsiella spp., Escherichia coli, Enterococci,* and *Anaerobic streptococci*,* *infecting an ischemic gallbladder [1-8]. These bacteria are gas-producing and increase the chance of gallbladder rupture by stressing the already compromised organ structure [3-4]. Gallbladder rupture can lead to rapid deterioration in clinical condition, as seen in this case.

Emphysematous cholecystitis can present insidiously due to the vague symptoms and spectrum of severity. Patients may present with acute right upper quadrant pain, fever, jaundice, nausea, and vomiting [1,2]. Additionally, patients may have a positive Murphy's sign on examination [2]. However, patients may deteriorate at a rate incongruent with their pain level with any delay in diagnosis and intervention [2,4]. In the case of our patient, she is a female under the age of 60 without predisposing medical history highlighting the vitality of imaging to diagnosis.

Abdominal ultrasound is often used as the initial imaging for detecting gallbladder disease and can be used to diagnose emphysematous cholecystitis. On ultrasound, gas may be detected in the gallbladder lumen, wall, or pericholecystic tissue and can be used to stage emphysematous cholecystitis [1,3,9-10]. In addition, emphysematous cholecystitis may present as an effervescent gallbladder on ultrasound with the appearance of multiple bubbles in the lumen or curvilinear gaseous artifacts known as the “ringdown effect” [1,3,9-10]. Both are diagnostic for emphysematous cholecystitis but may not be present in all cases. While abdominal ultrasound is a standard screening tool, the location of gas in the biliary tract secondary to emphysematous cholecystitis can impair imaging and lead to false negatives. The most sensitive and specific imaging tool for emphysematous cholecystitis is computerized tomography [3,10]. Computerized tomography revealing gas in the biliary tree is diagnostic for emphysematous cholecystitis and can provide further information on the location and extent of air collection determining the best options for intervention [3,9-10].

Further investigation for emphysematous cholecystitis may include a complete blood count to assess for leukocytosis, liver function tests in the setting of concurrent choledocholithiasis, and serum glucose for patients with diabetes mellitus [1,11].

The definitive treatment for emphysematous cholecystitis is emergent cholecystectomy [11]. The method used for cholecystectomy is dependent on the stage of emphysematous cholecystitis and the confirmation of complications [1,11]. If the patient has peritonitis, indications of gangrene or perforation, or imaging concerning pneumoperitoneum, an open cholecystectomy is indicated such as in our patient [1,11]. If there is no concern for these complications, the cholecystectomy may be performed robotically or laparoscopically with a low threshold for open conversion. If a patient is unable to tolerate anesthesia due to a clinical condition, a cholecystostomy can be performed as a stabilizing procedure while the patient is medically optimized for a later cholecystectomy [3-5,11].

Postoperative assessment should include a complete blood count analysis, a hepatobiliary iminodiacetic acid scan, and careful monitoring for any signs of retained common duct stones [1,11]. A hepatobiliary iminodiacetic acid scan should be used postoperatively to assess any leakage from the cystic duct stump [1,11].

## Conclusions

To conclude, this report outlines a case of a 57-year-old female who presented with upper abdominal pain, nausea, vomiting, leukocytosis, and a positive Murphy’s sign. The radiologic findings of mottled air in the gallbladder lumen, extraluminal air, and an elevated right hemidiaphragm confirm the diagnosis of perforated emphysematous cholecystitis. In instances where patients present with symptoms suspicious of acute cholecystitis, radiologic testing should be done early to determine if emergent surgery is necessary to avoid complications such as sepsis and shock. Following the operation on our patient, a complete blood count analysis and hepatobiliary iminodiacetic acid scan were done, both of which were unremarkable.
